# The synthetic cannabinoid WIN55,212-2 mesylate decreases the production of inflammatory mediators in rheumatoid arthritis synovial fibroblasts by activating CB_2_, TRPV1, TRPA1 and yet unidentified receptor targets

**DOI:** 10.1186/s12950-016-0114-7

**Published:** 2016-05-05

**Authors:** Torsten Lowin, Georg Pongratz, Rainer H. Straub

**Affiliations:** Funktionsbereich & Hiller Forschungszentrum für Rheumatologie, Life Science Center, University Hospital Duesseldorf, Merowingerplatz1A, 1. Etage, D-40225 Duesseldorf, Germany; Laboratory of Experimental Rheumatology and Neuroendocrine Immunology, University Hospital of Regensburg, D-93053 Regensburg, Germany

**Keywords:** Cannabinoid, Synovial fibroblasts, Cytokines, MMP, Proliferation, Arthritis

## Abstract

**Background:**

In rheumatoid arthritis (RA), synovial fibroblasts (SF) secrete large amounts of IL-6, IL-8 and matrix metalloproteinases (MMPs) which are crucial for cartilage destruction. RASFs are sensitive to the action of cannabinoids and they not only express cannabinoid receptors type I and II (CB_1_ and CB_2_) but also transient receptor potential channels type vanilloid (TRPV1) and ankyrin (TRPA1). The synthetic cannabinoid WIN55,212-2 mesylate (WIN) demonstrated strong anti-inflammatory effects in monocytes and synovial fibroblasts only in high concentrations in a non-cannabinoid receptor dependent manner. In this study we assessed the ability of WIN to modulate cytokine and MMP-3 production in SFs over a wide concentration range and identified specific receptor targets that mediate the effects of this synthetic cannabinoid.

**Methods:**

MMP-3, IL-6 and IL-8 were determined by ELISA. Adhesion was measured by the XCELLigence system. Proliferation was assessed by cell titer blue assays.

**Results:**

WIN significantly reduced TNF-induced IL-6, IL-8 and MMP-3 production in concentrations below 2 μM, while higher concentrations completely inhibited production of IL-6 and IL-8 but increased extracellular MMP-3 levels. The inhibitory effect at low concentrations (<2 μM) was independent on activation of either CB_1_ or CB_2_ but was attenuated by TRPV1 or TRPA1 inhibition in OASFs and RASFs. The effects of high concentrations of WIN on cytokine and MMP-3 production were decreased by the calcium chelating agent BAPTA, the AMPK activator metformin, the TRPA1 antagonist A967079 and the CB_2_ antagonist COR170. Furthermore, fetal calf serum content in culture media strongly influenced the efficacy of WIN at high concentrations. In addition, high concentrations of WIN also diminished SF adhesion and proliferation without altering cell viability whereas low concentrations promoted SF adhesion without any influence on proliferation.

**Conclusion:**

The synthetic cannabinoid WIN in low concentrations exhibits anti-inflammatory effects in synovial fibroblasts independent of CB_1_ and CB_2_ while CB_2_ and yet unidentified receptor targets are responsible for WIN effects in micromolar concentrations. Our results indicate a TRPV1/TRPA1 dependent mechanism of SF regulation that might be coupled to cellular energy status and calcium content.

## Background

Rheumatoid arthritis (RA) is a chronic inflammatory autoimmune disease characterized by joint inflammation and cartilage destruction [1]. The latter is mediated mostly by macrophages and synovial fibroblasts (SFs) which secrete matrix degrading enzymes, activate lymphocytes and invade cartilage [2, 3]. Although several therapeutic options are available for the treatment of RA, none of these specifically target SFs although they are a major contributor to the disease.

Besides its role in controlling neurotransmitter release, the endocannabinoid system influences several aspects of the immune response where it acts mostly immune-modulatory. Peripheral anti-inflammatory effects of (endo-) cannabinoids have been attributed to the activation of the cannabinoid receptor 2 (CB_2_) while CB_1_ is the major cannabinoid receptor in the central nervous system where it controls neurotransmitter release [4, 5]. The endocannabinoid arachidonylethanolamine (anandamide ;AEA) decreases proliferation and cytokine production of T-cells and this was dependent on activation of CB_2_ [6]. In collagen-induced arthritis in mice, elevation of the endocannabinoid tone by inhibition of degradation was protective via a CB_2_-dependent mechanism [7]. A similar protective effect was achieved using a synthetic CB_2_ agonist [5]. In vitro studies with isolated synovial fibroblasts also demonstrated anti-inflammatory effects of some synthetic cannabinoids albeit only in micromolar concentrations, possibly, not via classical cannabinoid receptors [8].

In this study, we investigated the mechanism of action of one of the most widely used CB_1_/CB_2_ agonists, WIN55,212-2 mesylate (WIN). It is demonstrated that not cannabinoid receptors but transient receptor potential channels (TRPs) vanilloid type 1 (TRPV1) and ankyrin (TRPA1) mediate the anti-inflammatory effects of WIN in physiological concentrations on rheumatoid arthritis synovial fibroblasts (RASFs) and osteoarthritis synovial fibroblasts (OASFs). In addition, we show that micromolar concentrations of WIN decrease cytokine production by activating CB_2_ and non-cannabinoid receptor targets. Furthermore, the effect of WIN on SF adhesion and proliferation were investigated.

## Methods

### Patients

In this study, 28 patients with long-standing RA fulfilling the American College of Rheumatology revised criteria for RA [9] and 56 patients with OA were included. The RA group comprised of 21 females and 7 males with a mean age of 61.1 years ±10.7 years; C-reactive protein was 7.0 mg/dl ± 8.59 mg/dl. In the RA group, 23 patients received non-steroidal anti-inflammatory drugs, 22 glucocorticoids, 11 methotrexate, 3 sulfasalazine and 2 biologicals. The OA group comprised of 31 females and 25 males with a mean age of 68.5 years ±9.2 years; C-reactive protein was 4.7 mg/dl ± 10.4 mg/dl. In the OA group, 45 patients received non-steroidal anti-inflammatory drugs. All patients underwent elective knee joint replacement surgery, and they were informed about the purpose of the study and gave written consent. The study was approved by the Ethics Committee of the University of Regensburg.

### Synovial fibroblast and tissue preparation

Synovial tissue samples from OA and RA were obtained immediately after opening the knee joint capsule, the preparation of which was recently described [10]. Pieces of synovial tissue of up to 9 cm^2^ were excised. One part of the tissue was cut, placed in protective freezing medium and stored at −80 °C until further use (Tissue Tek, Sakura Finetek, Zoeterwoude, The Netherlands). Another part was minced and treated with dispase I (Roche Diagnostics, Mannheim, Germany). Digestion was carried out for at least 1 h at 37 °C on a shaking platform. The resulting suspension was filtered (70 μm) and centrifuged at 300 g for 10 min. The pellet was then treated with erythrocyte lysis buffer (20.7 g NH_4_Cl, 1.97 g NH_4_HCO_3_, 0.09 g EDTA ad 1 l H_2_O) for 5 min and again centrifuged for 10 min at 300 g. The pellet was resuspended in RPMI-1640 (Sigma Aldrich, St. Louis, USA) with 10 % FCS. Cell number was calculated using a Neubauer cell counting chamber. A total of 100,000 cells were transferred to a 75 cm^2^ tissue culture flask. After overnight incubation, cells were supplemented with fresh medium.

### Stimulation of RA and OA synovial fibroblasts

To study cytokine and MMP-3 production, cells were stimulated in 2 % serum-containing RPMI-1640 medium with respective compounds 5 h prior to addition of TNF (10 ng/ml final concentration). Cell culture supernatants were used for ELISAs 24 h or 48 h after addition of compounds, respectively.

### TNF, IL-6 and IL-8 ELISA

Tests were conducted as described by the supplier (BD, OptEIA, Heidelberg, Germany). Tissue culture supernatants from SFs were diluted 1:13 (RASFs and OASFs) (for IL-6 and IL-8). Inter- and intraassay coefficient of variation was below 10 %.

### Matrix metalloproteinase-3 (MMP-3) ELISA

A total of 100 μl of tissue culture supernatants were transferred into a 96 well plate (NUNC, Langenselbold, Germany) and frozen at −20 degrees. Plates with supernatants were defrosted for 3 h at 37 degrees. Supernatants were removed and 1 % BSA in PBS was added for 1 h at room temperature to block unspecific binding. Then, anti-MMP3 (ab52915, abcam, Cambridge, UK) antibody was added for 1 h at room temperature (1:1000, diluted in PBS with 1 % BSA). After washing, secondary antibody (1:2000, goat anti-rabbit Poly-HRP, Fisher Scientific, Schwerte, Germany) was added for 1 h at room temperature. Authentic MMP-3 protein was obtained from R&D systems.

### Monitoring adhesion with the xCELLigence system

The xCELLigence system (Roche Applied Sciences, Mannheim, Germany) enables real time monitoring of cellular events by using impedance measurements across interdigitated micro-electrodes integrated on the bottom of tissue culture microtiter plates (E-plates, electrode plates). Impedance changes occur if cells attach to E-plates or change their size, shape and number due to specific treatments.

For adhesion experiments, E-plates (Roche) were coated for 1 h at room temperature with 100 μl 10 μg/ml fibronectin (BD, Heidelberg, Germany) in PBS. To block unspecific binding, 1 % bovine serum albumin in PBS was added for 30 min. A total of 2000–5000 pre-treated cells (see above) were added to the E-plates and xCELLigence was programmed to monitor adhesion every minute for 240 min. After 60 min of incubation, adhesion remained linear so that time point was chosen as the endpoint of adhesion measurement. Adhesion was quantified by averaging data from 1 min to 60 min of incubation. Untreated cells served as control and this value was set to 100 %.

### Statistical analysis

Statistical analysis was performed with SigmaPlot 12 (Systat Software Inc., San Jose, USA) and SPSS 20 (IBM, Armonk, USA). The statistic tests used are given in the figure legends. The significance level was *p* < 0.05.

## Results

### WIN reduces IL-6, IL-8 and MMP-3 production in RA and OA synovial fibroblasts

**Fig. 1:**
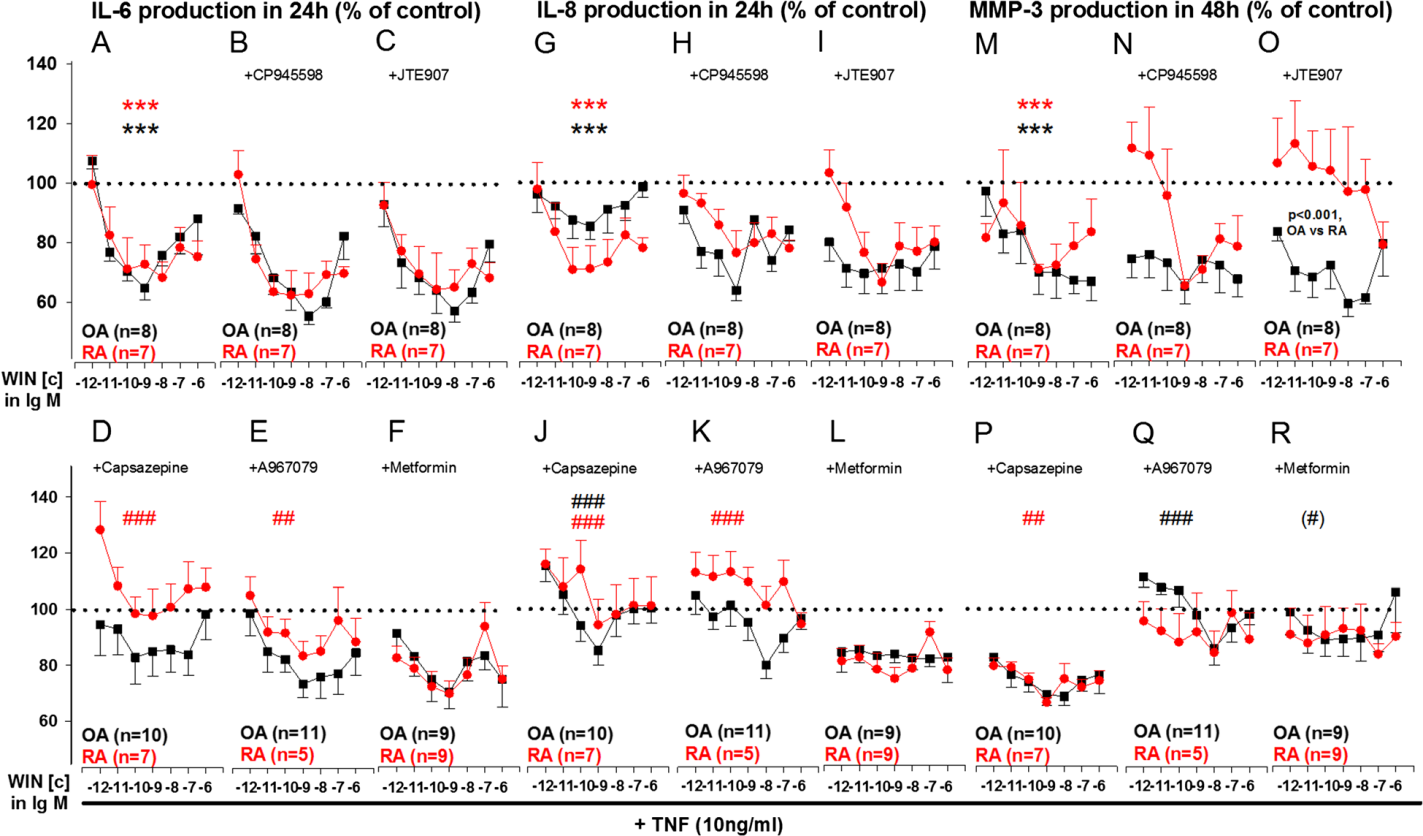
Influence of WIN55212-2 mesylate (WIN, 10^−12^M to 10^−6^M) with and without antagonists on TNF-induced IL-6 (**a**-**f**), IL-8 (**g**-**l**) and MMP-3 (**m**-**r**) production in RA and OA synovial fibroblasts. Cytokine/MMP-3 production was determined under hypoxic (1 % O_2_) conditions. ***p < 0.001 for comparisons vs control (TNF only, 100 %, dotted line). ###*p* < 0.001, ##*p* < 0.01 for comparisons vs WIN/TNF only w/o antagonists. The general linear model with Bonferroni correction was used for all comparisons. All data are given as mean ± sem. CB_1_ antagonist = CP945598, 1 μM; CB_2_ antagonist = JTE-907, 1 μM; TRPV1 antagonist = capsazepine, 1 μM; TRPA1 antagonist = A967079, 1 μM; AMPK activator = metformin, 10 μM

In OASFs and RASFs, WIN (from 10^−12^M to 10^−6^M) concentration-dependently reduced TNF-induced IL-6 (Fig. 1a), IL-8 (Fig. 1g) and MMP-3 (Fig. 1m) production with highest efficacy in low to medium nanomolar concentrations. This inhibitory effect was not modulated by either CB_1_ or CB_2_ antagonists (Fig. 1b, c, h, i, n, o) but was attenuated by TRPV1 and TRPA1 antagonism. In RASFs treated with WIN, the decrease in TNF-induced IL-6 and IL-8 production was blocked by the TRPV1 antagonist capsazepine (Fig. 1d, j) and by the TRPA1 antagonist A967079 (Fig. 1e, k). In OASFs, only IL-8 production was modulated by TRPV1 antagonism (Fig. 1j). Extracellular MMP-3 levels were further reduced by TRPV1 antagonism in WIN/TNF treated RASFs (Fig. 1p), while TRPA1 antagonism reversed the inhibitory effect of WIN on MMP-3 production in OASFs (Fig. 1q). The AMPK activator metformin only showed a trend towards modulation of WIN effects on MMP-3 in OASFs (p = 0.058). Average control values were 4.11 ng/ml ± 3.05 ng/ml (OASFs) and 3.82 ng/ml ± 2.68 ng/ml (RASFs) for IL-6, 4.14 ng/ml ± 4.00 ng/ml (OASFs) and 3.60 ng/ml ± 1.58 ng/ml (RASFs) for IL-8 and 47 ng/ml ± 16 ng/ml (OASFs) and 42 ng/ml ± 17 ng/ml (RASFs) for MMP-3.

### Micromolar concentrations of WIN completely inhibit cytokine production but increase extracellular MMP-3 levels

**Fig. 2:**
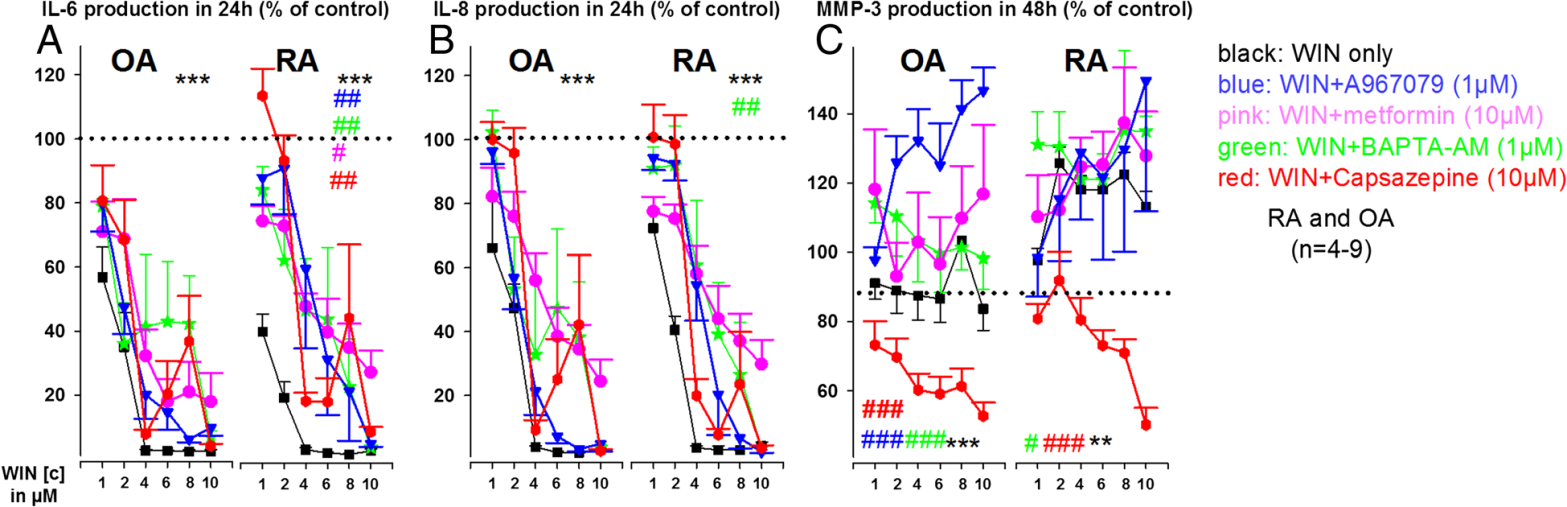
Influence of high concentrations of WIN55212-2 mesylate (WIN, 10^−6^M to 10^−5^M) with and without antagonists on TNF-induced IL-6 (**a**), IL-8 (**b**) and MMP-3 (**c**) production in RA and OA synovial fibroblasts. Cytokine/MMP-3 production was determined under hypoxic (1 % O_2_) conditions. ***p < 0.001 for comparisons vs control (TNF only, 100 %, dotted line). ###*p* < 0.001, ##*p* < 0.01, #*p* < 0.05 for comparisons vs WIN/TNF only w/o antagonists. The general linear model with Bonferroni correction was used for all comparisons. All data are given as mean ± sem. Calcium chelator = BAPTA-Am, TRPV1 antagonist = capsazepine, TRPA1 antagonist = A967079, AMPK activator = metformin

**Fig. 3:**
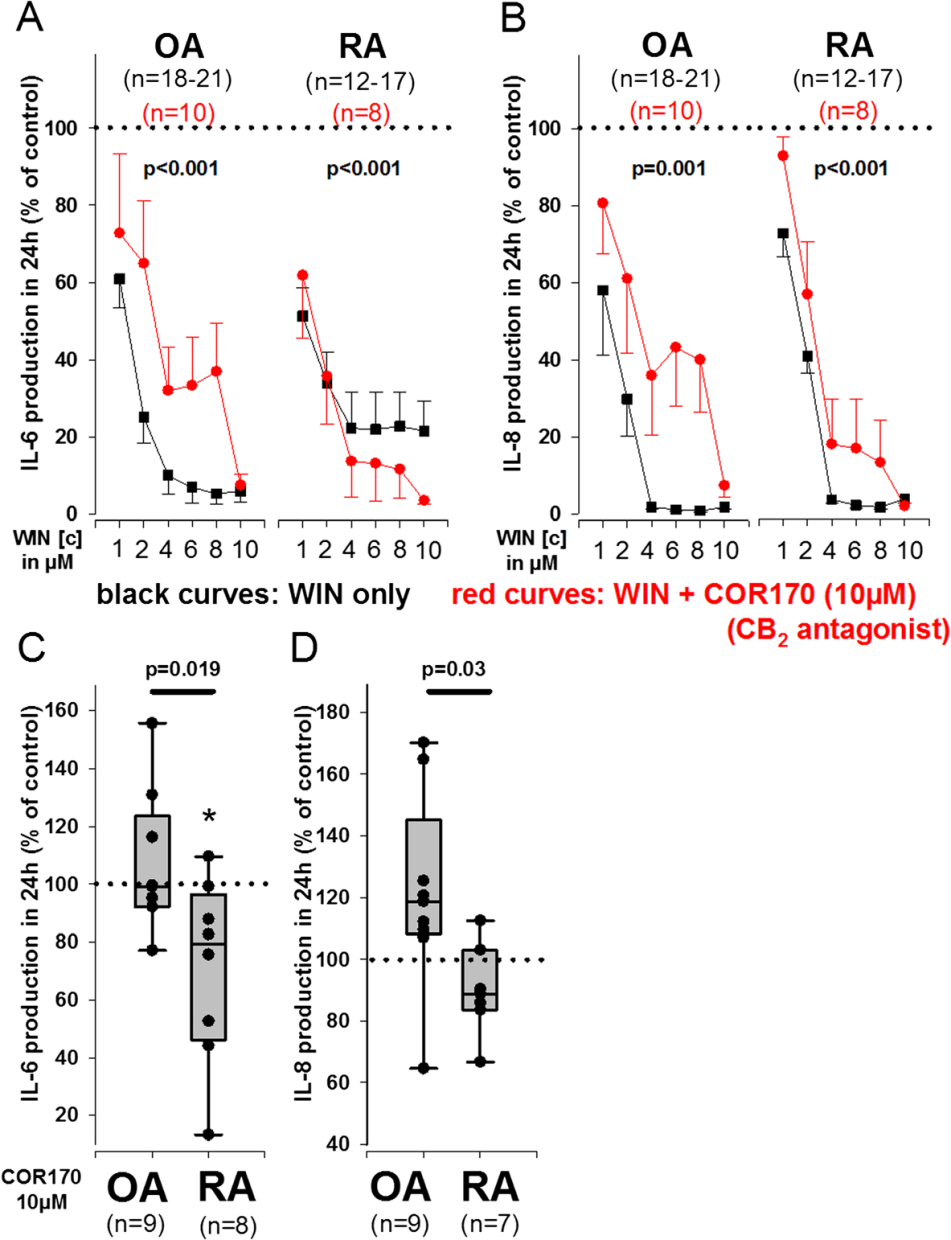
Influence of high concentrations of WIN55212-2 mesylate (WIN, 10^−6^M to 10^−5^M) and CB_2_ antagonist COR170 on TNF-induced IL-6 and IL-8 production in RA and OA synovial fibroblasts. **a**, **b** Modulation of WIN effects by CB_2_ antagonism on TNF-induced IL-6 and IL-8 production. **c**, **d** Impact of CB_2_ antagonist COR170 alone on IL-6 and IL-8 production in RASFs and OASFs. Cytokine production was determined under hypoxic (1 % O_2_) conditions. *p < 0.05 for comparisons vs control (TNF only, 100 %, dotted line). For comparisons versus control (100 %) the paired t-test was used; for comparisons between OA and RA (**c**, **d**), the t-test was used. The general linear model with Bonferroni correction was used for comparisons between WIN only and WIN together with CB_2_ antagonist (**a**, **b**). Data are depicted as mean ± sem. Corresponding *p*-values for comparisons are given in the graph. Data in C) and D) are visualized as box plots/vertical dot blots. The boundary of the box closest to zero indicates the 25th percentile, the line within each box indicates the median and the upper boundary of the box the 75th percentile. Error bars below and above the box indicate the 10th and 90th percentiles

WIN concentrations above 2 μM almost completely abolished IL-6 and IL-8 production in OASFs and RASFs (Fig. 2a, b), while extracellular MMP-3 levels were either unaltered (OASFs, Fig. 2c) or increased (RASFs, Fig. 2c). In OASFs, this strong inhibitory effect of WIN on IL-6 and IL-8 production was not altered by TRPV1/TRPA1 antagonism, AMPK activation by metformin or by removal of intracellular calcium by the chelating agent BAPTA-Am (Fig. 2a, b, OASFs). In RASFs, however, the inhibitory effect of WIN on TNF-induced IL-6 production was attenuated by i)TRPV1 and TRPA1 antagonism, ii) removal of intracellular calcium by BAPTA-Am and iii) the AMPK activator metformin (Fig. 2a, RASFs). IL-8 production was attenuated by BAPTA-Am only in RASFs (Fig. 2b, RASFs). Extracellular MMP-3 was reduced by addition of the TRPV1 antagonist capsazepine in OASFs and RASFs, while all other compounds (except A967079 in RASFs) increased extracellular MMP-3 levels (Fig. 2c). CB_1_ antagonism (1 μM CP945598, data not shown) did not significantly modulate the effects of high WIN concentrations, while the CB_2_ antagonist COR170 (10 μM) partially blocked the effects of WIN on IL-6 in OASFs but enhanced the effects of WIN on IL-6 production in RASFs. WIN-induced inhibition of IL-8 production was attenuated by COR170 in OASFs and RASFs (Fig. 3a, b). The CB_2_ antagonist/inverse agonist COR170 (10 μM) alone reduced IL-6 production only in RASFs (Fig. 3c) and it demonstrated different efficacy in RASFs and OASFs to modulate IL-6 and IL-8 production (Fig. 3c, d).

### Inhibitory effect of high WIN concentrations is partly dependent on media FCS content

**Fig. 4:**
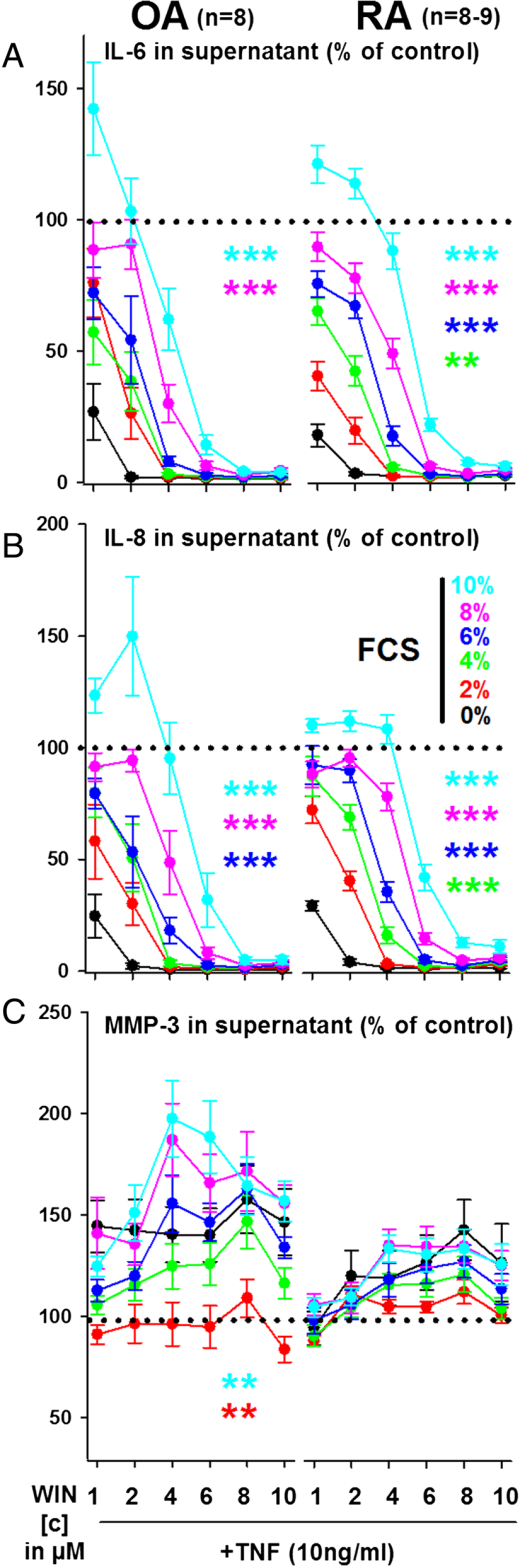
Impact of fetal calf serum (FCS) content on the effects of high concentrations of WIN55212-2 mesylate (WIN, 10^−6^M to 10^−5^M)) on TNF-induced IL-6 (**a**), IL-8 (**b**) and MMP-3 (**c**) production in RA and OA synovial fibroblasts. Cytokine/MMP-3 production was determined under hypoxic (1 % O_2_) conditions. ***p < 0.001, **p < 0.01, *p < 0.05 for comparisons vs WIN/TNF with 0 % FCS. The general linear model with Bonferroni correction was used for all comparisons. All data are given as mean ± sem

During above mentioned experiments, cells were cultured in medium containing 2 % fetal calf serum, which provides enough energy and growth factor input for cell maintenance but not to commence cellular proliferation (own observations). Therefore, the efficacy of high concentrations of WIN with varying amounts of FCS in culture medium was investigated. In OASFs and RASFs, the efficacy of WIN in modulating IL-6 and IL-8 production was highly dependent on FCS content. While the inhibitory effect of WIN was most pronounced without FCS, increasing FCS to 10 % significantly reduced its inhibitory capacity on TNF-induced IL-6 and IL-8 production (Fig. 4a, b). However, in concentrations above 6 μM, WIN abrogated cytokine production almost completely regardless of medium FCS content in OASFs and RASFs. Extracellular MMP-3 was only modulated by FCS in OASFs, with high FCS content increasing its levels (Fig. 4c, OASFs).

### WIN influences adhesion to fibronectin and proliferation of OASFs and RASFs

**Fig. 5:**
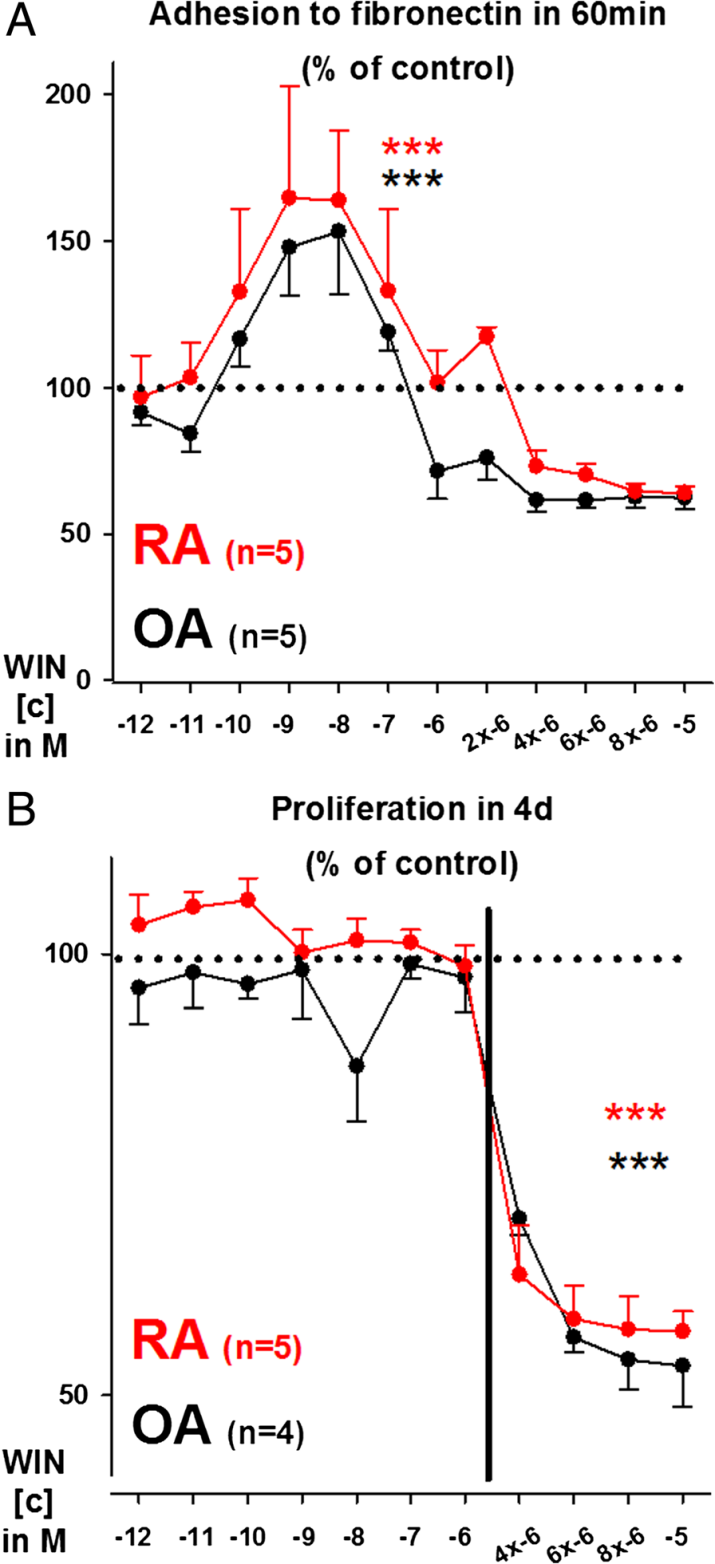
Influence of WIN55212-2 mesylate (WIN, 10^−12^M to 10^−5^M) on adhesion under normoxia and proliferation under hypoxia of OA and RA synovial fibroblasts. **a** Impact of WIN on OASF and RASF adhesion to fibronectin. **b** Impact of WIN on OASF and RASF proliferation. The general linear model with Bonferroni correction was used for all comparisons. The dotted line represents the control level without (tissue culture medium only) which was set to 100 %. Fig. 4a, b: Data are given as mean ± sem. The boundary of the box closest to zero indicates the 25th percentile, the line within each box indicates the median and the upper boundary of the box the 75th percentile; WIN data are given as median. Error bars below and above the boxes indicate the 10th and 90th percentiles. Fig. 4a: ***p < 0.001 for comparisons vs control. Fig. 4b: ***p < 0.001 for comparisons vs control, (WIN 2 μM to 10 μM only)

Cannabinoids not only influence the production of inflammatory mediators but also modulate cell functional parameters such as adhesion and proliferation. We have previously shown a stimulatory effect of CB_1_ and CB_2_ agonism on SF adhesion to fibronectin [11]. Therefore the ability of WIN to modulate SF adhesion was investigated. In OASFs and RASFs, WIN significantly modulated adhesion to fibronectin in a bell-shaped manner (Fig. 5a). While concentrations up to 100nM stimulated adhesion, higher concentrations decreased adhesion to fibronectin (Fig. 5a). Similar results were obtained when proliferation was investigated with WIN up to 1 μM being without significant influence (Fig. 5b). WIN concentrations above 1 μM, however, completely halted cell proliferation in OASFs and RASFs (Fig. 5b). Lactate dehydrogenase levels in the supernatant, a marker for cell death, remained unaltered for at least 48 h (data not shown).

### Treatment with high WIN concentrations changes cell morphology of SFs

**Fig. 6:**
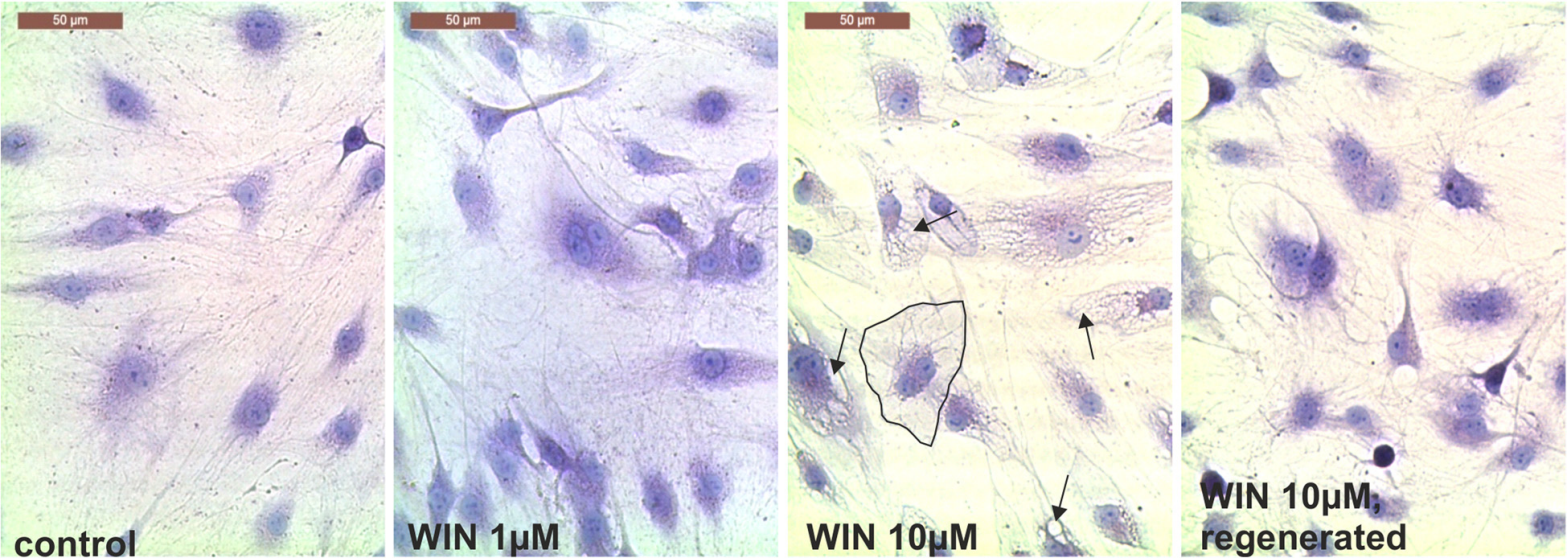
Cell morphologic changes of RA synovial fibroblasts after 48 h incubation with WIN (0 μM, 1 μM, 10 μM). Arrows indicate vacuolization; the black rimmed cell is an example of sharp edged cell bodies in contrast to unstimulated control fibroblasts. Regenerated = WIN wash-off after 48 h and supplementation with fresh medium containing 10 % FCS without WIN

Since high concentrations of WIN seem to impair OASF and RASF function, cell morphologic changes upon WIN treatment were investigated (Fig. 6). After 24 h, cell shape and size was altered upon treatment with various WIN concentrations (1 μM – 10 μM) and 2 % FCS (data not shown). These alterations were pronounced after 48 h and WIN concentrations above 2 μM induced changed cell shape from spread out with lots of filopodia to sharp-edged with a condensed nucleus and extensive vacuolation (Fig. 6). This effect was highly dependent on the presence of WIN, since wash-off and replacement of culture medium with fresh medium containing 10 % FCS fully reversed the effect within 24 h (Fig. 6).

## Discussion

In this study the anti-inflammatory effects of WIN in physiological (<1 μM) and high μM concentrations in RASFs and OASFs were investigated. It was demonstrated that WIN in concentrations below 1 μM decreases cytokine and MMP-3 production via a TRPV1 and TRPA1 dependent pathway independent of cannabinoid receptors type I and II (CB_1_ and CB_2_). This inhibitory effect of WIN was much enhanced at concentrations above 4 μM and was modulated only weakly by TRPV1/TRPA1 antagonism suggesting additional WIN targets activated at high concentrations. In OASFs but not RASFs, the effects of micromolar WIN concentrations were partially inhibited by CB_2_ antagonism. High WIN concentrations also demonstrated to impair SF adhesion and proliferation. The observed effects of high WIN concentrations were reduced by increasing FCS content in culture medium and cell morphologic changes induced by WIN were reversed by wash-off and replacement of culture medium without WIN.

Studies regarding the effects of synthetic cannabinoids on SFs were conducted by Selvi and colleagues who showed a cannabinoid receptor independent reduction of IL-6 and IL-8 production by high concentrations of synthetic cannabinoids CP55,940 and WIN but this study did not employ physiological concentrations of both compounds [12]. Two more recent studies by Gui and Fukuda employing different synthetic CB_2_ agonists claimed that CB_2_ activation is responsible for the reduction of IL-6 and IL-8 production in SFs, but both studies did not include antagonists [5, 8]. Furthermore the compounds used in these studies were effective only in very high concentrations (comparable to the high concentrations of WIN used in this study), suggesting off-target effects since their Ki values are in the low nanomolar range. Here we provide evidence that the effects of low WIN concentrations on TNF-induced IL-6, IL-8 and MMP-3 production were dependent on activation/desensitization of TRPV1 and TRPA1. The ability of WIN to modulate TRPA1 channel activity has been described in trigeminal neurons where WIN activates TRPA1 which in turn modulates TRPV1 channel activity via intracellular crosstalk [13, 14]. We found WIN to be effective at concentrations as low as 10nM, somewhat contradicting the Ki values reported for TRPA1 activation [15]. Since the binding site for WIN at TRPA1 is intracellular it might be possible that WIN is accumulated in the cytoplasm by binding to fatty acid binding proteins. This phenomenon has already been described for the endocannabinoid anandamide and plant cannabinoids delta9-tetrahydrocannabinol and cannabidiol [16].

In concentrations above 4 μM, we found WIN to completely block cytokine production. This is line with observations from other groups who showed similar effects in SFs but also in monocytes/macrophages [12, 17]. The latter study demonstrated a site specific effect of WIN since the enantiomer WIN55,212-3 mesylate was ineffective. Our study suggests an influence of WIN on intracellular calcium levels in SFs since BAPTA-Am, a calcium chelating agent, and TRPA1 antagonism (both treatments lower calcium) attenuated the inhibitory effect on TNF-induced cytokine production. Interestingly, CB_2_ antagonism partially reversed the inhibitory effects of micromolar WIN concentrations on IL-6 production in OASFs but not RASFs and although IL-8 production was attenuated by COR in OASFs and RASFs, the magnitude of inhibition was greater in OASFs. This might be due to altered intracellular signaling or desensitization of CB_2_ in RASFs. Previous studies already found specific hypomethylation patterns in RASFs supporting their characteristic “transformed” phenotype [18]. One of these changes might directly or indirectly impact CB_2_ signaling pathways.

In concentrations above 8 μM, the effects of WIN were no longer inhibited by CB_2_ antagonism, suggesting a different cellular target. Data from Bloom et. al demonstrate that WIN and other cannabinoids alter membrane polarization and many off-target effects of cannabinoids are only evident at concentrations that cause membrane perturbations [19]. This suggests that WIN and other cannabinoids alter the lipid environment of other receptors and thereby influence their accessibility to respective ligands [20]. Furthermore, the activity of adenylyl cyclase is enhanced by changes in membrane ordering counteracting the inhibitory effect of classical cannabinoid receptors on cAMP. [21]. In this study, we showed that the induction of autophagy by the AMPK activator metformin attenuated the effects of WIN on TNF-induced IL-6 production in RASFs, suggesting cellular energy perturbations. The observed increase in TNF-induced MMP-3 after treatment with high WIN concentrations is in contrast to observations in U937 macrophages, where WIN decreases extracellular MMP-9 [17].

We found fetal calf serum (FCS) content in culture medium to modulate the efficacy of WIN up to concentrations of 6 μM, with high FCS being protective. This effect was also evident in breast cancer cells where serum free conditions enhanced the potency of WIN threefold [22]. The protective effect of serum might derive from enhanced WIN binding to serum proteins thereby slowing exposure to the compound.

In previous studies we found CB_1_ and CB_2_ agonism to support SF adhesion to fibronectin [11]. In concentrations up to 100nM, WIN also increased adhesion of RASFs and OASFs, likely via activation of classical cannabinoid receptors. In concentrations above 1 μM however, adhesion was decreased reflecting studies by Zhao that showed decreased adhesion of macrophages after WIN treatment [23]. Furthermore the beneficial effects of WIN in a chronic model of multiple sclerosis were partially mediated by down-regulating adhesion molecules VCAM-1 and ICAM-1 [24]. Interestingly, adhesion was maximally stimulated at WIN concentrations that were most effective in reducing IL-6 and IL-8 production (10^−9^M/10^−8^M). Conflicting results have been published as to whether CB_2_ activation enhances or decreases adhesion. While 2-AG and WIN increased adhesion of HL-60 macrophage-like cells to fibronectin, monocyte-endothelial cell adhesion was decreased by CB_2_ activation [25, 26]. This might depend on different adhesion molecules involved in these studies. Our results obtained with adhesion of SFs to fibronectin confirmed results of Gokoh et. al. Data from the literature suggest that integrin-driven adhesion is enhanced, but adhesion via VCAM1 and ICAMs is reduced by CB_2_ agonism [11, 26, 27]. Furthermore, adhesion might be influenced by the ligand used and biased agonism has been reported for CB_1_ and other GPCRs [28, 29]. Proliferation was modulated in a similar fashion with μM concentrations of WIN being inhibitory. This effect has been described in breast cancer cells and hepatocarcinoma cells albeit the latter study attributed the effect to PPAR-γ activation [22, 30]. Interestingly, other cannabinoids also provided non-cannabinoid receptor mediated anti-proliferative effects similar to those of WIN [31].

Changes in adhesion and proliferation with high dose WIN treatment was accompanied by changes in cell morphology leading to sharp edged cell bodies, condensed nuclei and extensive vaculolation. This is in line with observations in mantle cell lymphoma cells which responded similar to WIN [32]. In this study, the authors did not find any apoptotic markers but attributed cell death to paraptosis. This confirms our results since we did not detect any increases in lactate dehydrogenase (marker for general cell death) or caspase-3/7 (marker for apoptosis) but an increase in propidium iodide binding (marker for general cell death). High concentrations of WIN might mediate paraptosis by changes in membrane fluidity, since this might disrupt cellular processes required for cell survival [19–21].

## Conclusion

In this report we demonstrated anti-inflammatory effects of the synthetic cannabinoid WIN in low and high concentrations. While low WIN concentrations mediated effects via TRP channels, high concentrations were fully inhibitory on cell function and this was mediated via a site specific but yet unidentified receptor target. For therapeutic use, identification of this low-affinity WIN site of action might pose an attractive target especially when WIN is administered directly in the joint to achieve high local concentrations. Besides inhibiting cell function via its low affinity binding site, WIN also activates cannabinoid receptors 1 and 2 which have been shown to reduce (arthritic) inflammation [5, 33]. Furthermore, this study demonstrated anti-inflammatory effects via modulation of TRP channels by WIN. Together, inactivation of TRPs and activation of cannabinoid receptors might also reduce the sensation of pain, which further underlines the potential of WIN in the treatment of chronic inflammation.

## Abbreviations

AEA: arachidonylethanolamide, anandamide
CB_1_: cannabinoid receptor type 1
CB_2_: cannabinoid receptor type 2
IL-6: interleukin-6
IL-8: interleukin-8
MMP-3: matrix metalloproteinase 3 (stromelysin)
OA: osteoarthritis
RA: rheumatoid arthritis
SF: synovial fibroblasts
TNF: tumor necrosis factor
TRPA1: transient receptor potential ankyrin 1
TRPV1: transient receptor potential vanilloid 1
WIN: WIN55212-2 mesylate (*R*)-(+)-[2,3-Dihydro-5-methyl-3-(4-morpholinylmethyl)pyrrolo[1,2,3-*de*]-1,4-benzoxazin-6-yl]-1-naphthalenylmethanone mesylate)
